# ^125^I Radiotherapy combined with metronomic chemotherapy may boost the abscopal effect, leading to complete regression of liver metastasis in an SCLC patient with a 58.5-month OS: a case report

**DOI:** 10.3389/fonc.2023.965166

**Published:** 2023-04-27

**Authors:** Linlin Lu, Yu Wang, Lei Li, Lan Yu, Li Liu, Baozhen Qu, Xiaotao Zhang

**Affiliations:** ^1^ Qingdao Cancer Prevention and Treatment Research Institute, Affiliated Qingdao Central Hospital of Qingdao University, Qingdao Cancer Hospital, Qingdao, China; ^2^ Department of Oncology, Affiliated Qingdao Central Hospital of Qingdao University, Qingdao Cancer Hospital, Qingdao, China; ^3^ Department of Oncology, Shandong Provincial Qianfoshan Hospital, Shandong University, Jinan, China

**Keywords:** SCLC, abscopal effect, metronomic chemotherapy, permanent iodine-125 seeds implantation, liver metastases

## Abstract

The liver is the most common and lethal metastatic site in patients with extensive-stage small-cell lung cancer (ES-SCLC), and median survival with current standard treatment is only 9–10 months from diagnosis. Clinical observations show that a complete response (CR) is extremely rare in ES-SCLC patients with liver metastasis. Moreover, to the best of our knowledge, complete regression of liver metastasis induced by the abscopal effect, boosted primarily by permanent radioactive iodine-125 seeds implantation (PRISI), combined with a low-dose metronomic temozolomide (TMZ) regimen, has not been recorded. Here, we present the case of a 54-year-old male patient who developed multiple liver metastases from ES-SCLC after multiple lines of chemotherapy. The patient was given partial PRISI therapy (two out of six tumor lesions; 38 iodine-125 seeds in one dorsal lesion and 26 seeds in one ventral lesion), which was combined with TMZ metronomic chemotherapy (50 mg/m^2^/day, days 1–21, every 28 days). The abscopal effect was observed for 1 month after PRISI treatment. After about 1 year, all the liver metastases had completely disappeared, and the patient experienced no relapse. The patient eventually died of malnutrition caused by a non-tumor intestinal obstruction and had an overall survival of 58.5 months after diagnosis. PRISI combined with TMZ metronomic chemotherapy might be considered a potential therapy to trigger the abscopal effect in patients with liver metastases.

## Introduction

Small-cell lung cancer (SCLC) is marked by its exceptionally high proliferation, early metastasis, and poor prognosis. The 5-year overall survival (OS) rate remains dismal, around 7%–10%, mainly because of the high risk of distant metastasis (1, 2). Among all the common metastatic sites, such as the liver, bones, brains, lungs, and adrenal glands, the liver is the most common site of metastasis, and metastasis to the liver, alone or in combination with metastasis to other organs, is associated with the worst outcomes (3–6). There were no important therapeutic clinical advances for over three decades (7, 8); a subset of patients have derived benefit from immunotherapy in recent years, but patients with liver metastases have not (9).

Radiation is a highly effective local treatment for tumor lesions. It works primarily by damaging the DNA inside cancer tissues. Spontaneous regression of tumors outside the irradiated field (abscopal effect) is rare but has been occasionally observed (10). However, the abscopal effect, induced by permanent radioactive iodine-125 seeds implantation (PRISI), has not been reported. Metronomic chemotherapy is designed to maintain low, but active, concentrations of chemotherapeutic drugs over prolonged periods of time without causing serious toxicities (Y. L. 11). As has been reported, metronomic chemotherapy can promote tumor regression not only by inducing anti-angiogenesis but also by increasing latent antitumor immune responses (12, 13).

The aim of this report is to present the case of a patient with extensive stage- (ES-)SCLC who showed an unusual liver abscopal effect after receiving PRISI combined with temozolomide (TMZ) metronomic chemotherapy. This resulted in a sustained complete response (CR) and long-term survival. The patient died of malnutrition caused by a non-tumor intestinal obstruction 15 months after PRISI.

## Case presentation

Here we present the case of a 54-year-old male patient who had previously been diagnosed with SCLC and received multiple lines of chemotherapy regimens, including etoposide and cisplatin (EP), irinotecan and cisplatin (IP), and paclitaxel and cisplatin (TP). He had also received radiotherapy of the chest wall, the right supraclavicular region, the thoracic vertebrae, and the right adrenal gland from December 2013 to August 2015 at another hospital, and was assessed as having a partial response. Unfortunately, he experienced multiple relapses, and presented to our clinic in October 2015.

**Figure 1:**
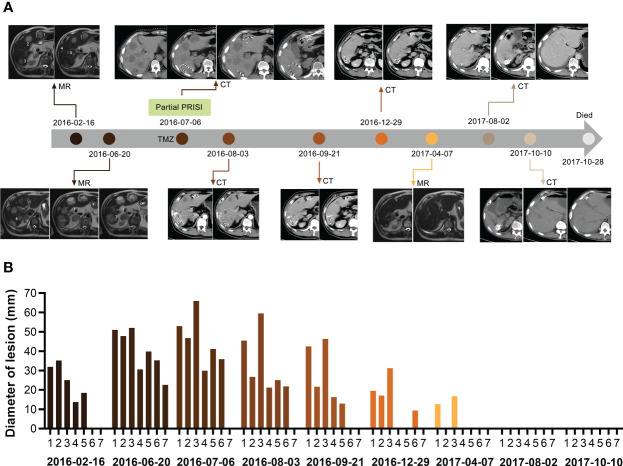
Course of the complete regression of the patient’s multiple liver metastases. **(A)** MR/CT scans of the patient’s liver before and after partial PRISI and treatment with the low-dose metronomic TMZ regimen. **(B)** Diagram showing the progressive change in the size of tumor lesions. Lesion numbers labeled on scans correspond to the same lesion numbers in the diagram. PRISI, permanent radioactive iodine-125 seeds implantation; TMZ, temozolomide.

Before treatment, a magnetic resonance (MR) scan was carried out, and revealed multiple metastases to the right axilla, bilateral supraclavicular lymph nodes, the right seventh rib, and the left ilium. His disease progressed and, after treatment with two cycles of EP and one cycle of single paclitaxel chemotherapy, a MR scan revealed multiple liver metastases (**Figure 1A**, 2016–02–16). No tumor response was observed after completion of two cycles of combined chemotherapy with albumin-bound paclitaxel and carboplatin, but a new right-side pleural metastasis was found. He then received radiotherapy to the right chest wall (50 Gy in 25 fractions) and achieved a CR. Specimens of the initial tumor were analyzed for sequencing mutations. The tumor was found to be SCLC and to contain no proven drug-sensitive gene mutations. In any event, treatment targeting the programmed cell death 1 ligand 1 (PD-1/L1) had not yet been approved in China in 2015.

The patient’s liver metastases significantly progressed, with two masses measuring > 50 mm in diameter and five metastases > 20 mm in diameter: individually, 50.91mm, 47.75mm, 52mm, 30.58mm, 39.86mm, 35.26mm, 22.61mm in diameter (**Figures 1A, B**, 2016–06–20). The patient complained of severe pain in the upper right abdomen. After a multidisciplinary discussion, we decided to apply computer tomography- (CT-)guided PRISI as a salvage treatment. The two lesions chosen for treatment were those that would require the shortest path for the implantation surgery, aiming to minimize secondary damage to the patient’s liver. In detail, the patient underwent permanent implantation of ^125^I seeds (measuring 4.5 × 0.8 mm, half-life of 60.2 days, photon energy spectrum 27–35 keV, radioactive activity of 0.7 mCi) in two of the liver metastases (one dorsal and one ventral) in July 2016. The prescription dose at the target volume was 120 Gy. We implanted 38 seeds in the dorsal lesion and 26 seeds in the ventral lesion (**Figures 1A**, 2016–07–06).

In addition, a low-dose metronomic TMZ regimen (50 mg/m^2^/day, days 1–21, every 28 days) was instituted before PRISI local therapy as a low-toxicity systemic therapy. This was also done to prevent secondary brain metastasis, because clinical studies have shown that 40%–50% of SCLC patients develop brain metastases after completion of palliative chemotherapy (this was not a standard of care use of TMZ in SCLC).

Follow-up CT in August and September 2016 revealed that all of the patient’s liver lesions were significantly reduced, including the four liver lesions not treated with PRISI and also those outside the ^125^I seeds radiation field, which means that abscopal regression was observed (**Figures 1A, B**, 2016–08–03, 2016–09–21).

CT and MR scans taken between December 2016 and April 2017, i.e., 5–9 months after implantation, showed that nearly all of the patient’s metastatic nodules, whether or not treated with PRISI, had undergone CR. The remaining two lesions had achieved partial response (PR), shrinking in diameter in one case from 52.86 mm to 12.62 mm and in the other case from 65.92 mm to 16.69 mm (**Figures 1A, B**, 2016–12–29, 2017–04–07).

Extraordinarily, all liver nodules had achieved CR by around 1 year after PRISI therapy (**Figure 1**, 2017–08–02). Follow-up results 13–15 months post PRISI therapy showed continued CR of liver metastases (**Figures 1A, B**, 2017–08–02 and 2017–10–10).

Unfortunately, the patient died of malnutrition caused by a non-tumor intestinal obstruction on 28 October 2017.

## Discussion and conclusions

Radiotherapy has traditionally been reserved for the palliation of symptoms in patients with advanced disease, including in those who have poor responses to chemotherapy. Brachytherapy has been used for the clinical treatment of malignant tumors worldwide for many years (14), including for hepatocellular carcinoma (HCC) (15, 16), lung cancer (17, 18), prostate cancer, pancreatic cancer, pulmonary carcinoma, oral and maxillofacial tumors, and head and neck malignant neoplasms (19; K. 15, 20–29). Its therapeutic efficacy has been reported to be promising.

PRISI has been used in the liver, and there are reports of PRISI increasing OS in HCC patients after curative resection and in patients with metastatic liver cancer (25; K. 15, 30). In addition, PRISI has been reported to result in a high rate of CR and PR in patients with advanced unresectable HCC (26). Li et al. (16) suggested that palliative surgery plus PRISI is an appropriate therapeutic option for patients with large (diameter >5 cm) HCC tumors. In this case, we performed partial PRISI (in only one dorsal nodule and one ventral nodule) as salvage treatment to reduce tumor-related clinical symptoms and improve the patient’s quality of life. To our surprise, abscopal regression was observed in the liver metastatic lesions that were not treated with ^125^I seeds. Normally, the abscopal effect is driven by external beam radiotherapy (EBRT), which is prescribed to control the disease (10). The median reported radiation dose is 31 Gy (range: 0.45–60.75 Gy) with a median dose of 3 Gy per fraction (range: 0.15–26 Gy) (10). In this case, the prescription dose at the target volume was 120 Gy. Brachytherapy with interstitial implantation of radioactive seeds can achieve a high dose within the target area but the irradiation is sharply attenuated with distance (radiation diameter of 1.7 cm) (30), with the original dose reduced to 1% at 5 cm from the source. However, in this patient, multiple liver metastasis nodules first shrank and then eventually exhibited CR. In fact, the four lesions not treated with PRISI, i.e., which were not located inside the ^125^I seeds radiation field, within 1 month exhibited abscopal regression in the same way as the as two lesions implanted with ^125^I seeds. Impressively, follow-up MR and CT scans at 5 months after treatment showed PR or CR of multiple liver metastatic lesions, and subsequent MR and CT scans at 13–15 months’ follow-up showed complete resolution. The patient’s quality of life, as self-reported, improved significantly.

Studies suggest that immunological mechanisms play a key role in this rare phenomenon (31, 32). When radiation damage tumor cells to liberate tumor-associated antigens (TAAs) like necrotic and apoptotic tumor cells and debris. Increasing and diversity of TAAs stimulate tumor-specific immune response, antigen-presenting cells (APCs) engulf these TAAs and then activate CD8+ T cells to attack the tumor tissue (32). Irradiated tumor cells may also release cellular danger-associated molecular patterns (DAMPs) and cytokines that enhance the migration of immune cells (33).

On the other hand, we speculate that our metronomic TMZ regime also played a crucial role in this case, combining with ^125^I brachytherapy to boost the abscopal effect. Metronomic chemotherapy is designed to maintain low, but active, concentrations of chemotherapeutic drugs over prolonged periods of time without causing serious toxicities (Y. L. 11). Metronomic chemotherapy can promote tumor regression not only by inducing anti-angiogenesis but also by selectively depleting immunosuppressive cells such as myeloid-derived suppressor cells (MDSCs) and regulatory T cells (Tregs) and by increasing latent antitumor immune responses (C. A. 11–13, 34). Banissi et al. found that a low-dose metronomic TMZ regimen reduced the Treg/CD4^+^ ratios in the spleen of tumor-bearing rats (35). In our case, a low-dose metronomic TMZ regimen (50 mg/m^2^/day, days 1-21, every 4 weeks) was instituted before PRISI to prevent secondary brain metastasis, as clinical studies have shown that 40%–50% of SCLC patients develop brain metastases after completion of palliative chemotherapy. As shown from the clinical results, this patient still benefited from metronomic TMZ chemotherapy even after multiline treatment, including EP, IP, and TP. Besides, without grade 3-4 adverse events and fewer incidents of treatment interruption during the whole course of treatment. In addition, metronomic chemotherapy is known to induce tumor cells to release TAAs, initiating a T-cell anti-tumor response in the same way as radiation. Moreover, signals released by killed tumor cells would have had an impact on phagocytosis and/or antigen processing, or the maturation and trafficking of dendritic cells (13, 36–38). Taken together, the liver immune microenvironment might have been changed by PRISI and metronomic TMZ treatment in this patient. Regrettably, we did not undertake pathology or laboratory studies to measure cytokines and immune cell subsets and, therefore, have no relevant laboratory data to confirm the postulated mechanisms, mentioned above, in this case.

To our knowledge, this is the first study to report that PRISI boosts the abscopal effect in an ES-SCLC patient with multiple metastases. The metronomic TMZ regime may also help to generate abscopal regression in this therapeutic process. Although more clinical and laboratory trials are needed to elucidate the mechanism, PRISI combined with metronomic chemotherapy is a potential salvage treatment and could be used to control tumor metastases with few complications.

## Data availability statement

The original contributions presented in the study are included in the article/**Supplementary Material**. Further inquiries can be directed to the corresponding author.

## Ethics statement

Written informed consent was obtained from the individual(s) for the publication of any potentially identifiable images or data included in this article.

## Author contributions

Conceptualization: LLu and XZ. Treatment decision-making and discussions: XZ, LLi, YW, LY, and LLiu. Data collection and analysis: LLu, BQ, and XZ. Manuscript writing: LLu. Final approval of manuscript: XZ. All authors contributed to the article and approved the submitted version.
