# Supplementary figures and images for: 125I Radiotherapy combined with metronomic chemotherapy may boost the abscopal effect, leading to complete regression of liver metastasis in an SCLC patient with a 58.5-month OS: a case report
Source: Front Oncol. 2023 Apr 27;13:965166. doi: 10.3389/fonc.2023.965166 (PMC10172687; doi:10.3389/fonc.2023.965166)

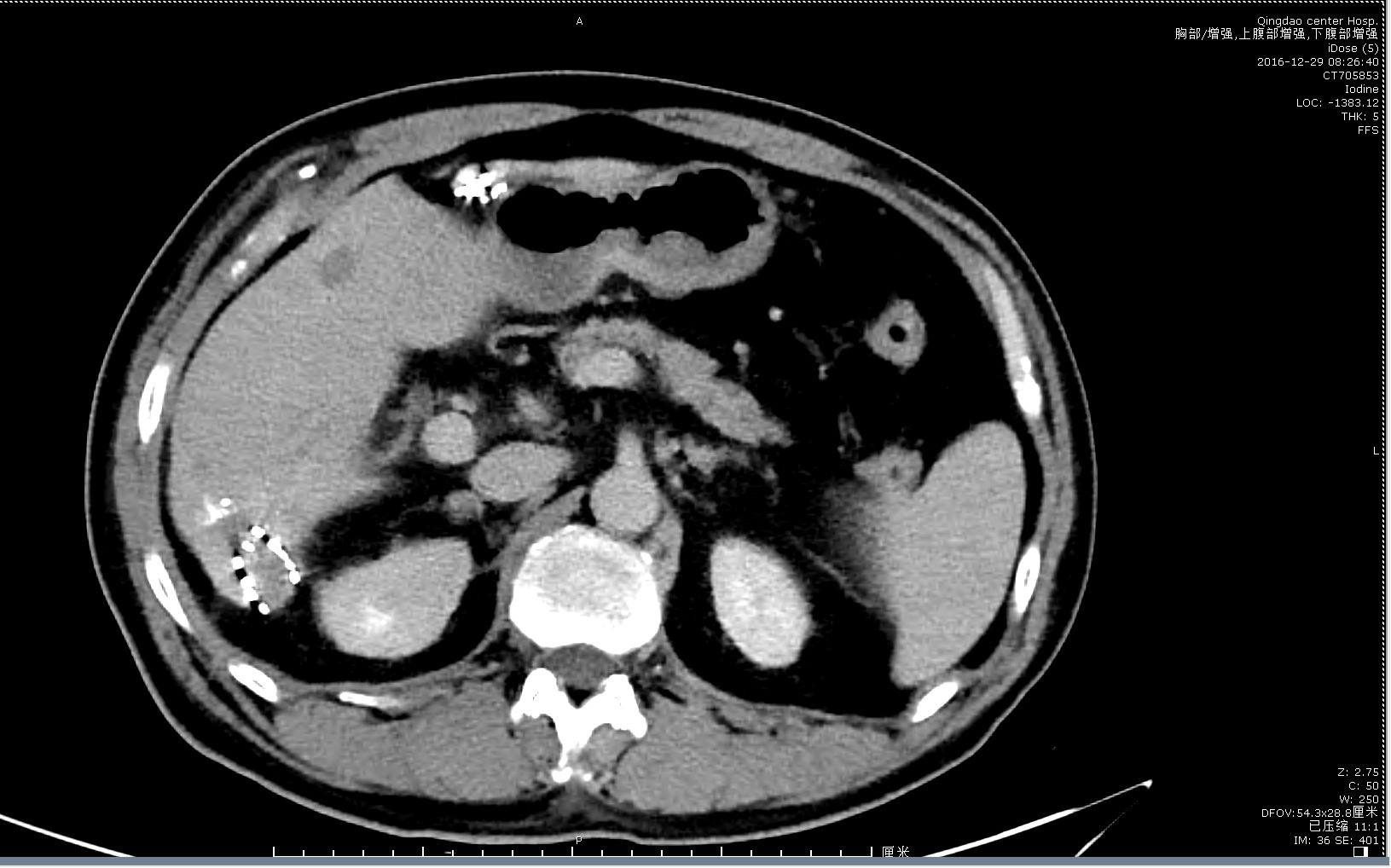

Supplement: Supplementary file 1 [file DataSheet_1.zip › original image/2016-12-29 Fig1.JPG]

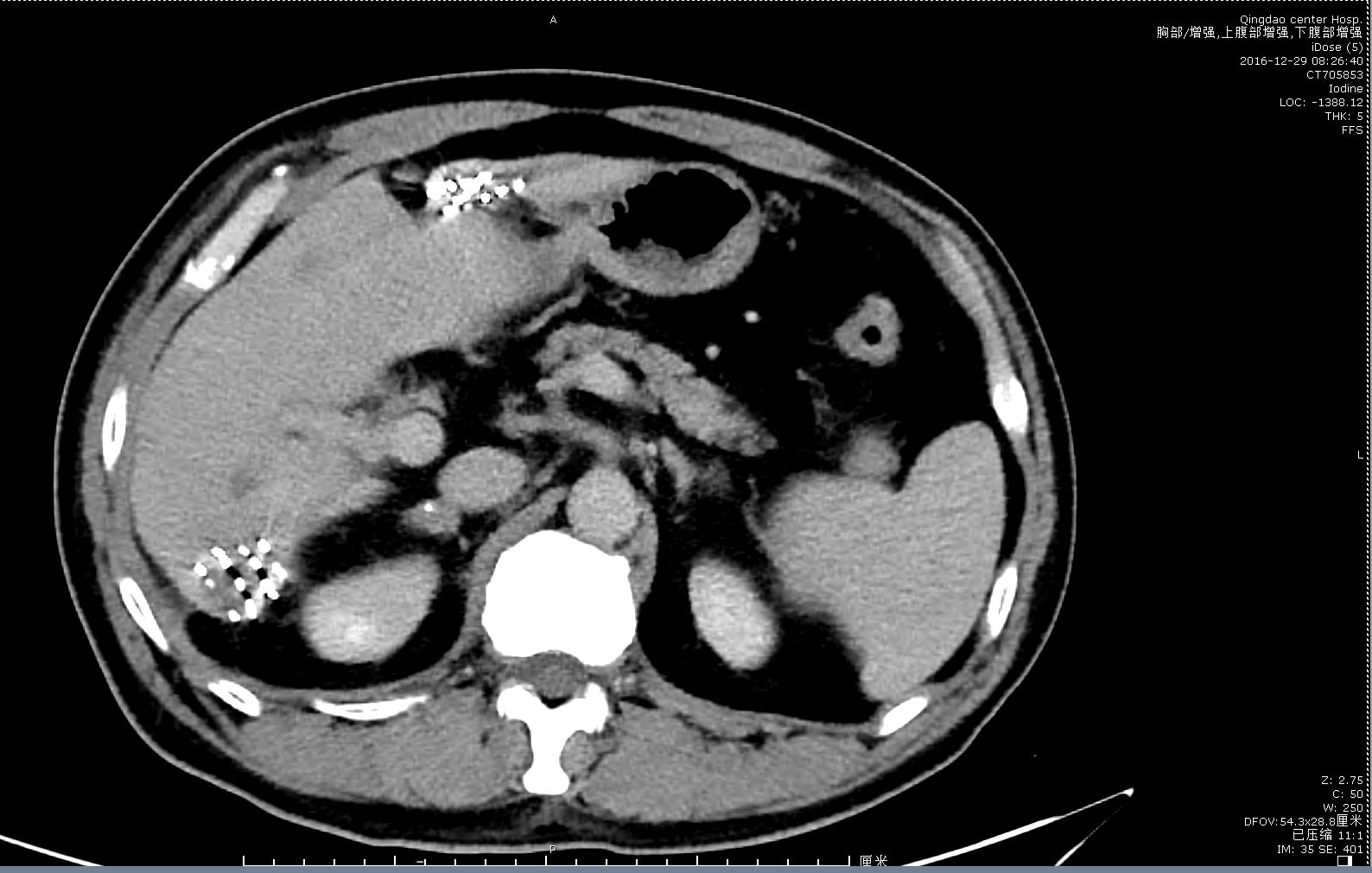

Supplement: Supplementary file 1 [file DataSheet_1.zip › original image/2016-12-29 Fig2.JPG]

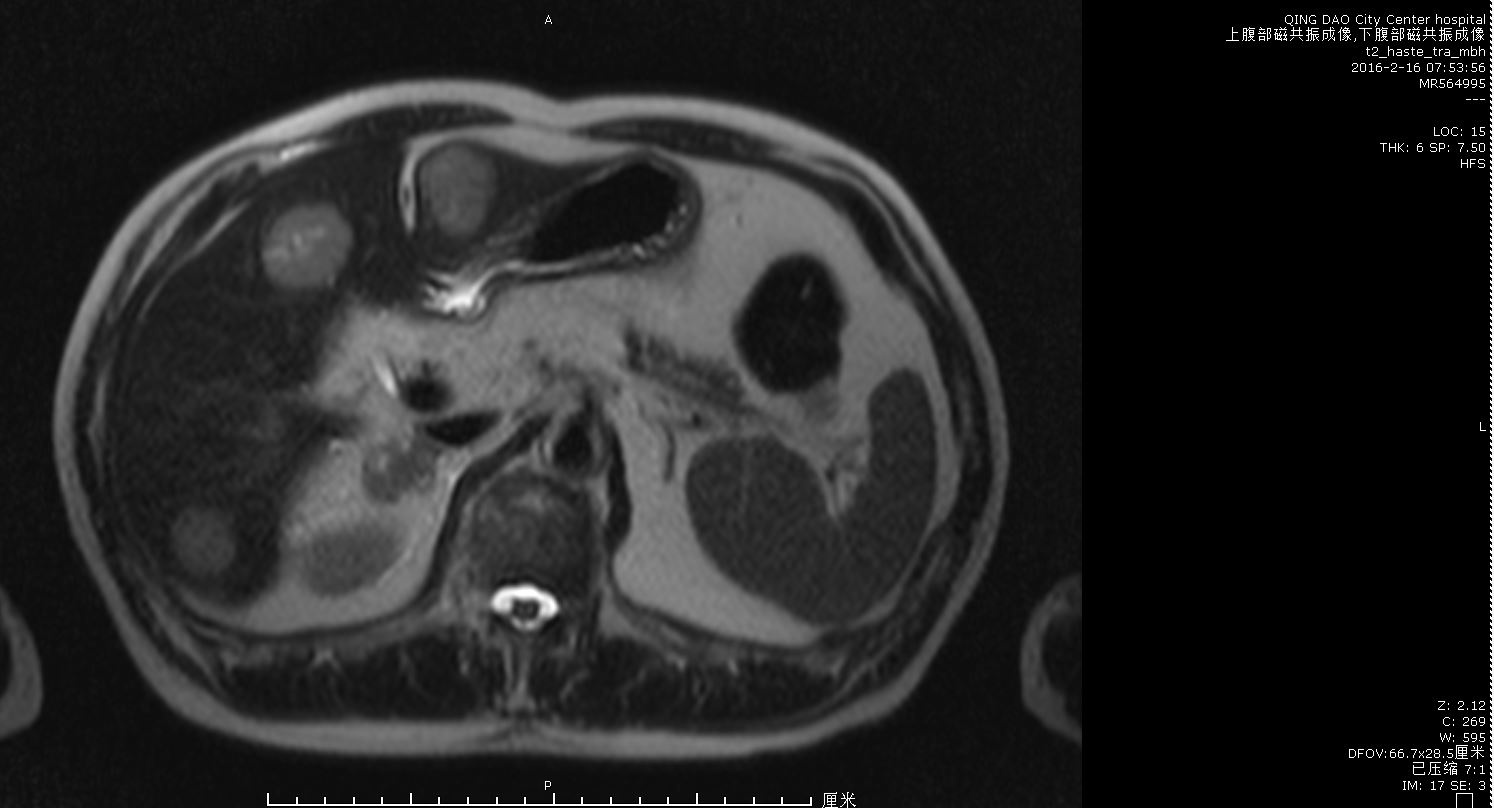

Supplement: Supplementary file 1 [file DataSheet_1.zip › original image/2016-2-16 Fig1.JPG]

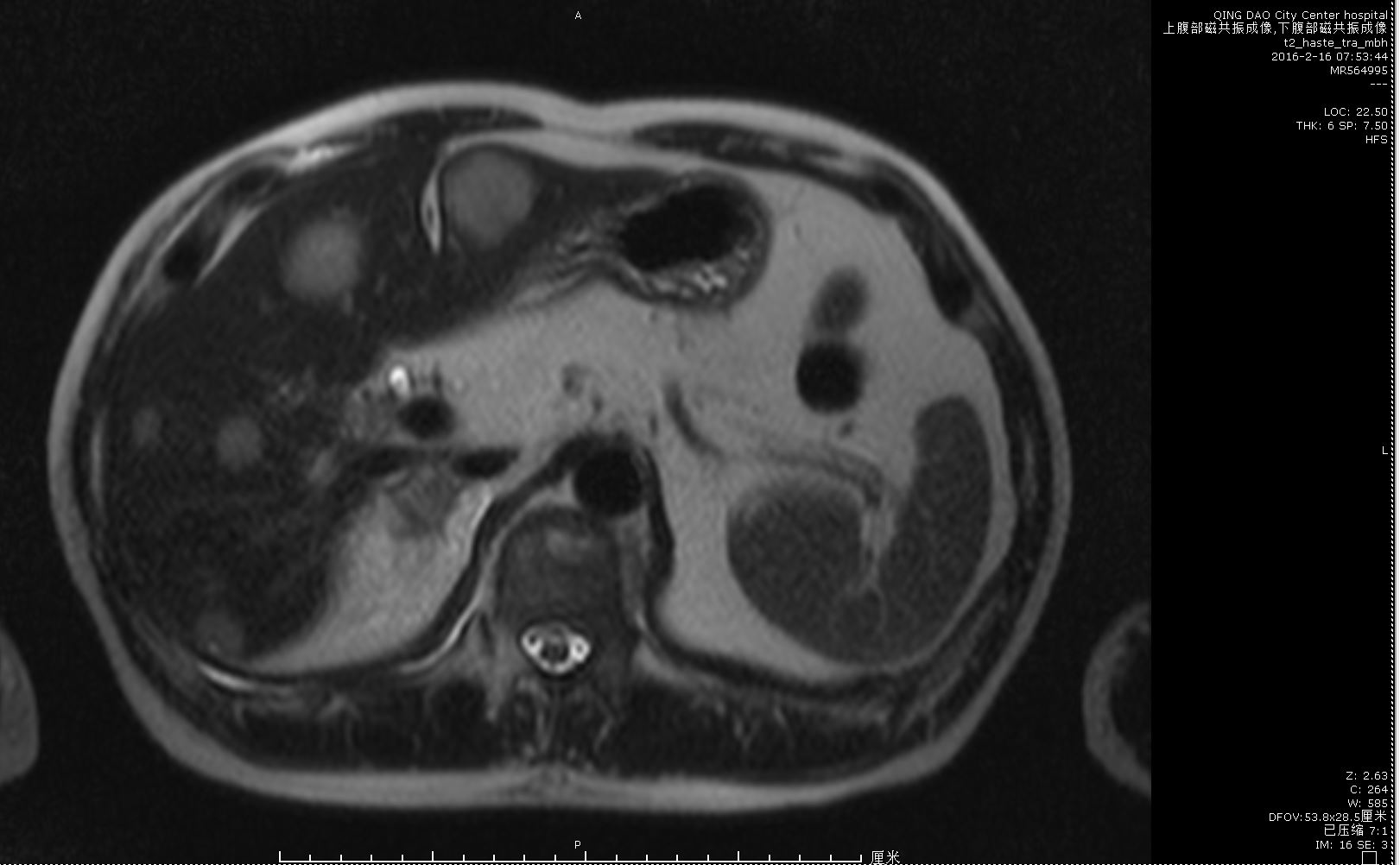

Supplement: Supplementary file 1 [file DataSheet_1.zip › original image/2016-2-16 Fig2.JPG]

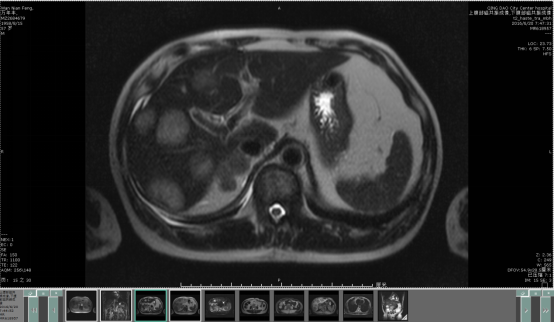

Supplement: Supplementary file 1 [file DataSheet_1.zip › original image/2016-6-20 Fig1.jpg]

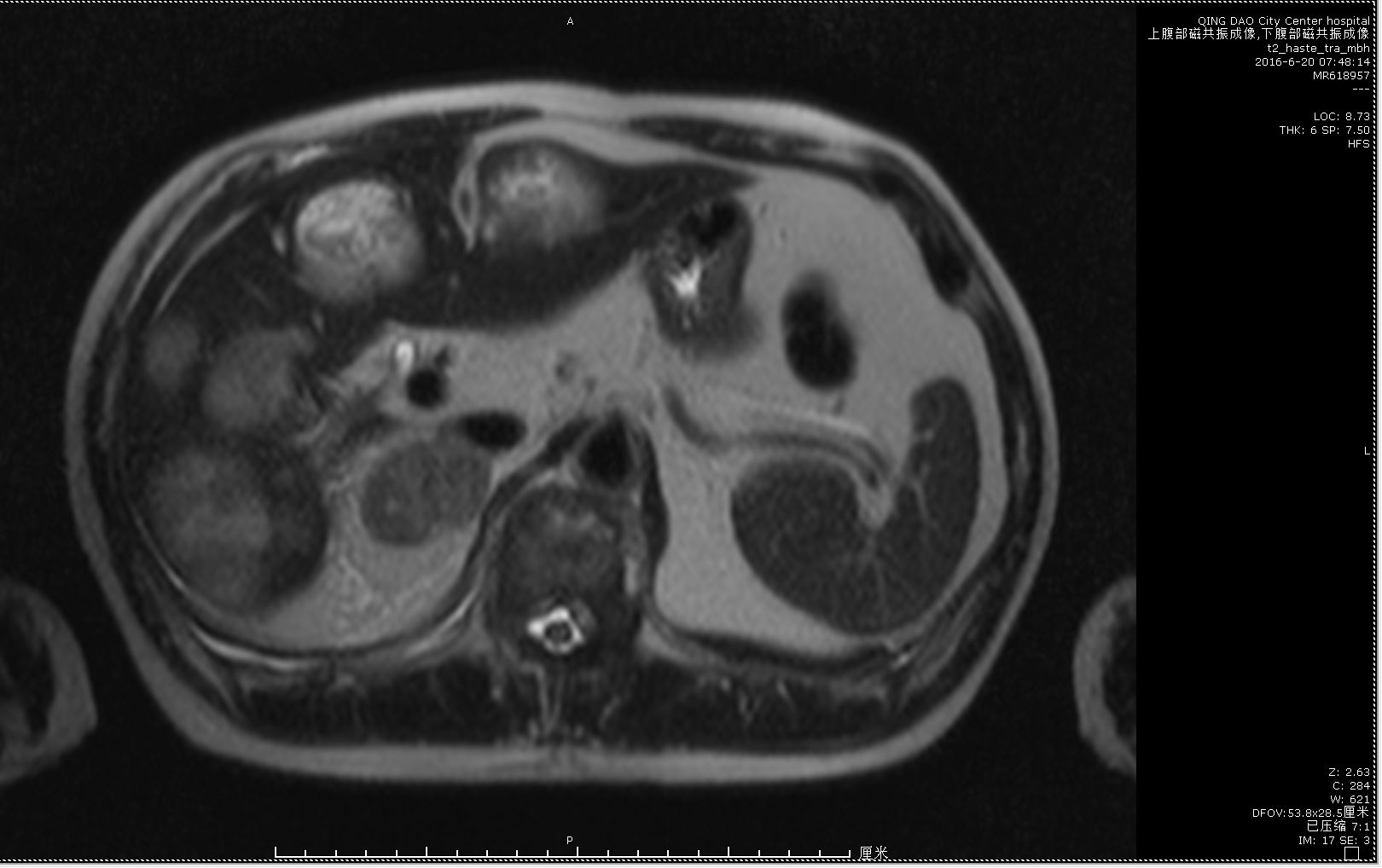

Supplement: Supplementary file 1 [file DataSheet_1.zip › original image/2016-6-20 Fig2.JPG]

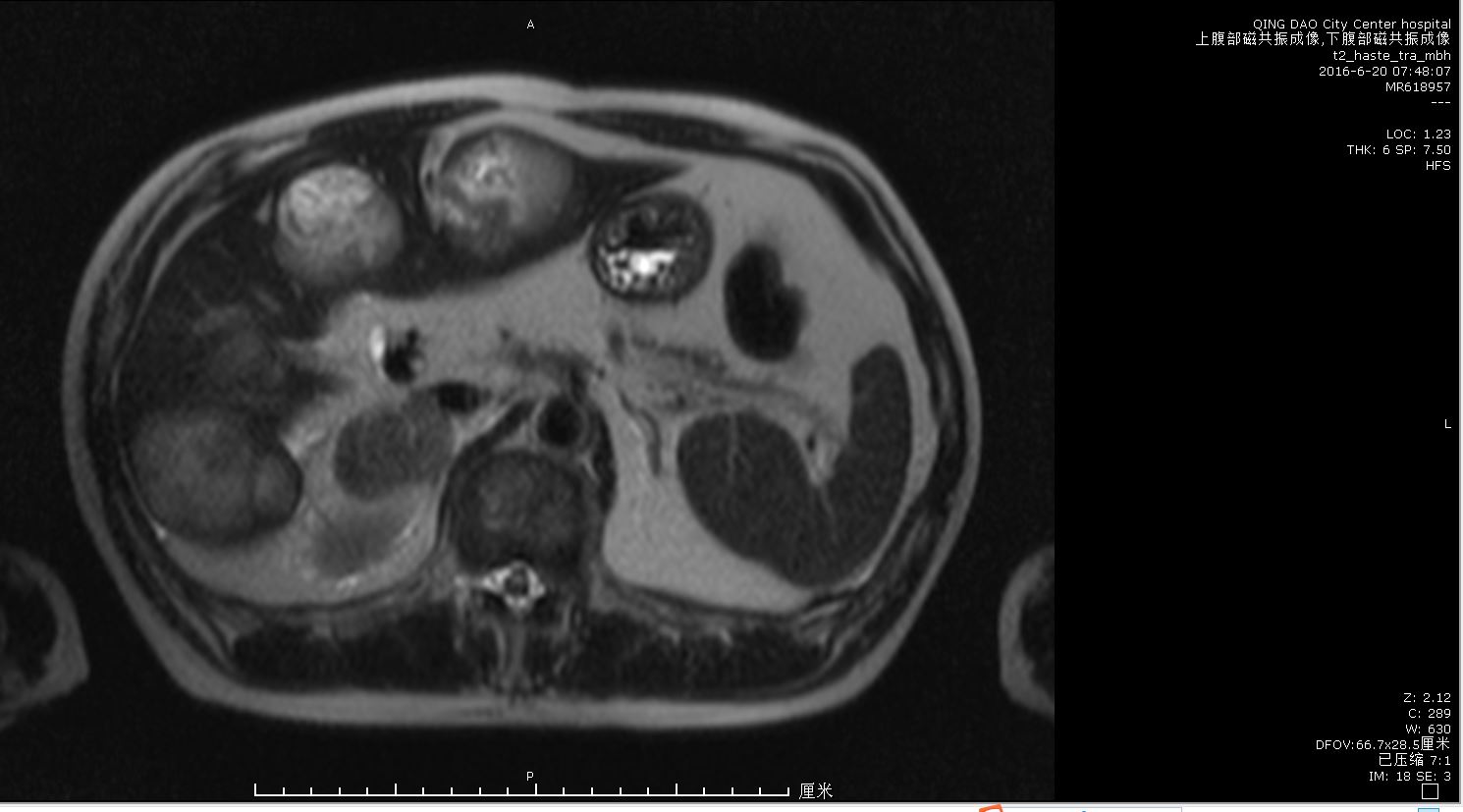

Supplement: Supplementary file 1 [file DataSheet_1.zip › original image/2016-6-20 Fig3.JPG]

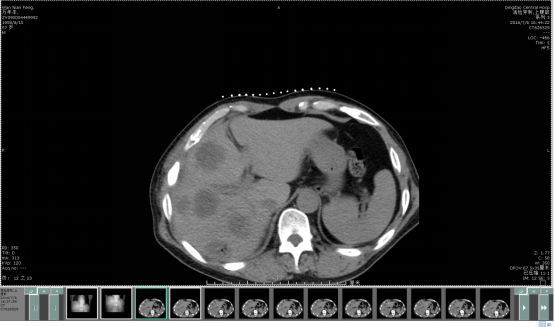

Supplement: Supplementary file 1 [file DataSheet_1.zip › original image/2016-7-6 Fig1.jpg]

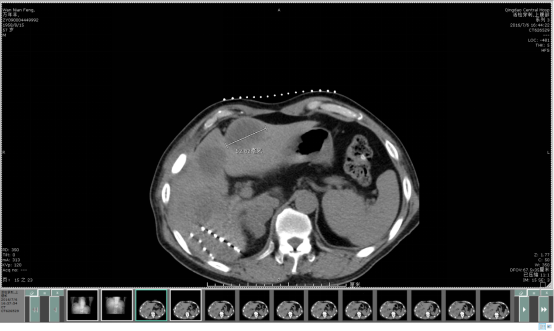

Supplement: Supplementary file 1 [file DataSheet_1.zip › original image/2016-7-6 Fig2.jpg]

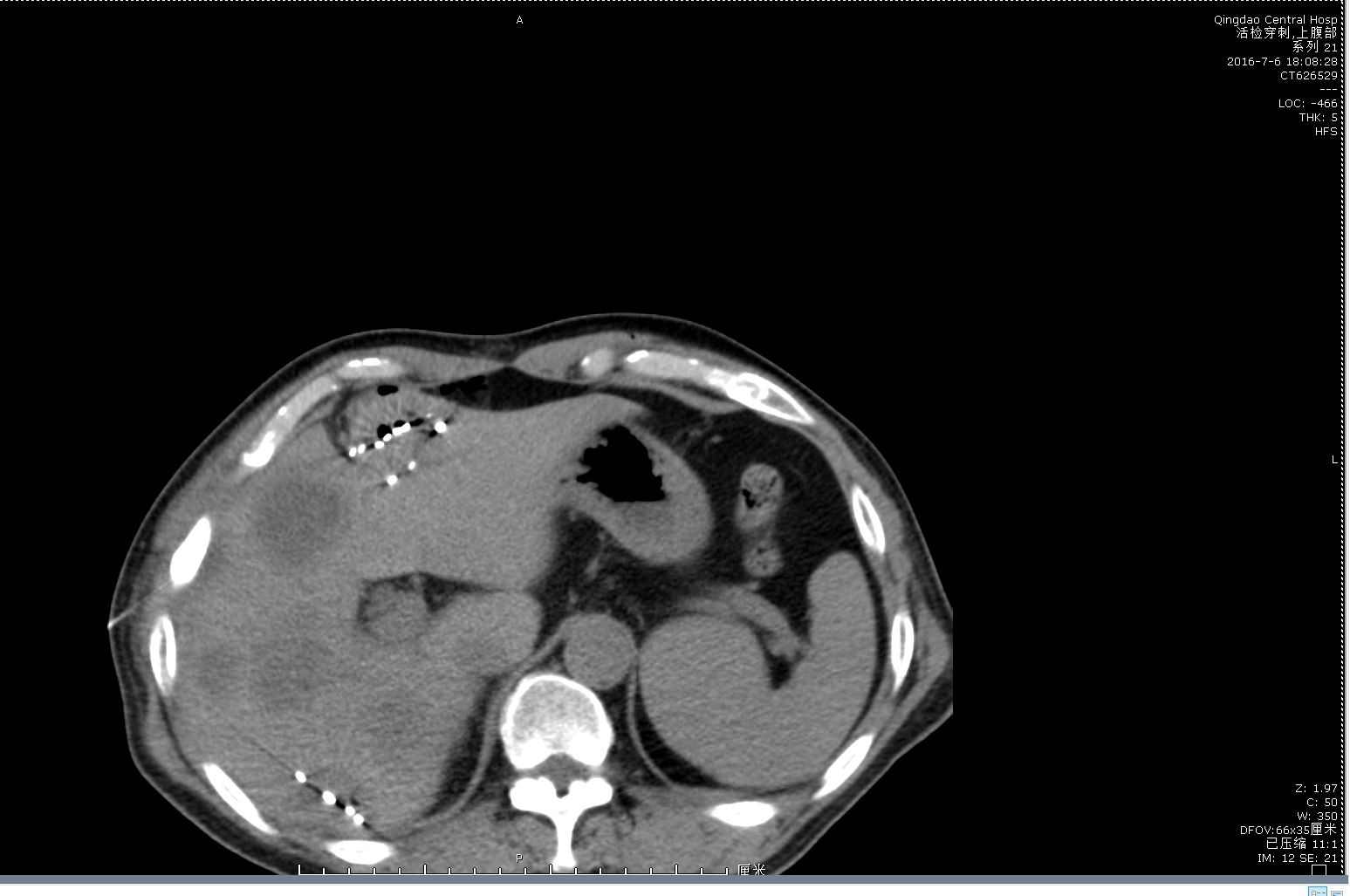

Supplement: Supplementary file 1 [file DataSheet_1.zip › original image/2016-7-6 Fig3.JPG]

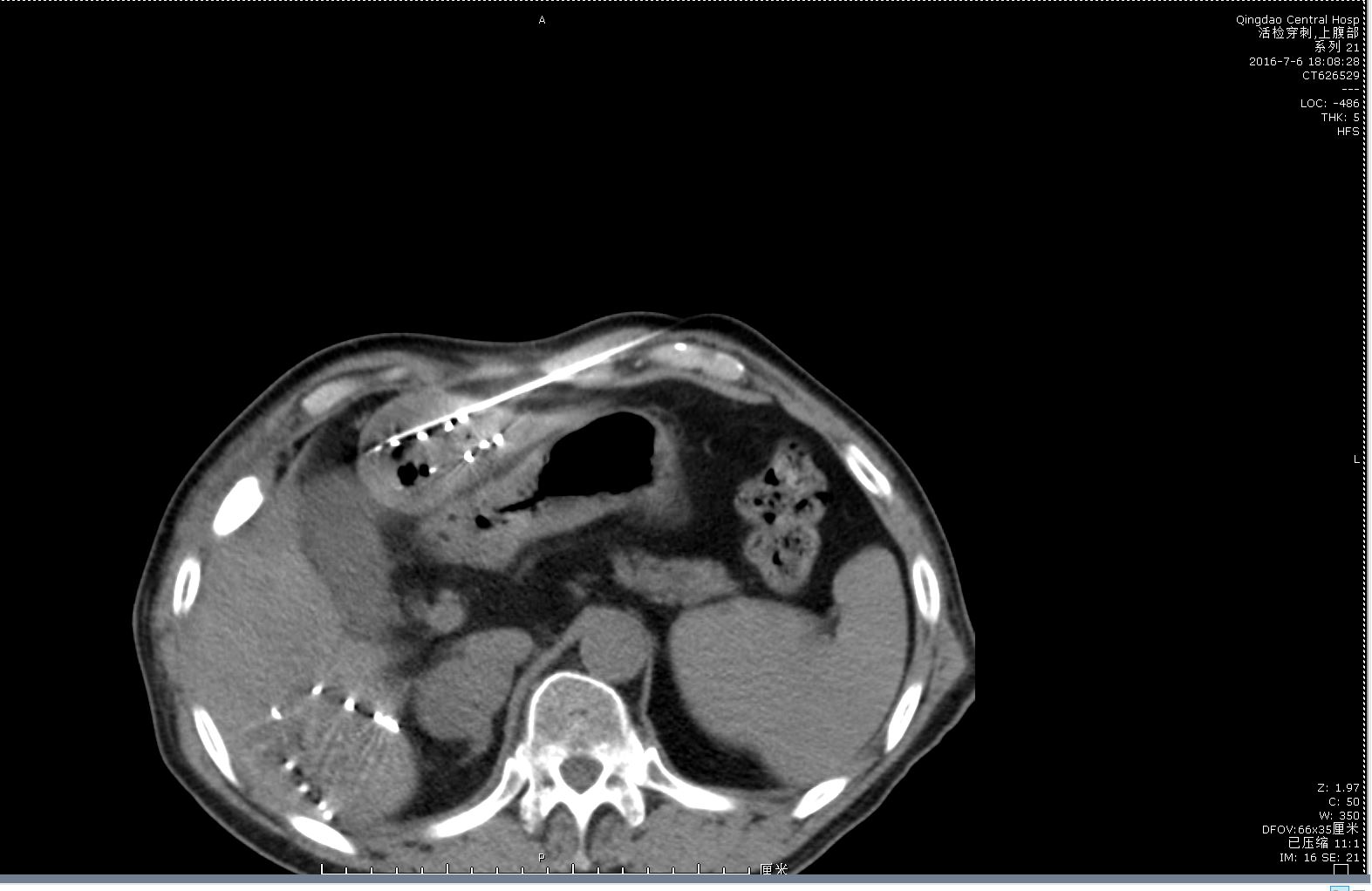

Supplement: Supplementary file 1 [file DataSheet_1.zip › original image/2016-7-6 Fig4.JPG]

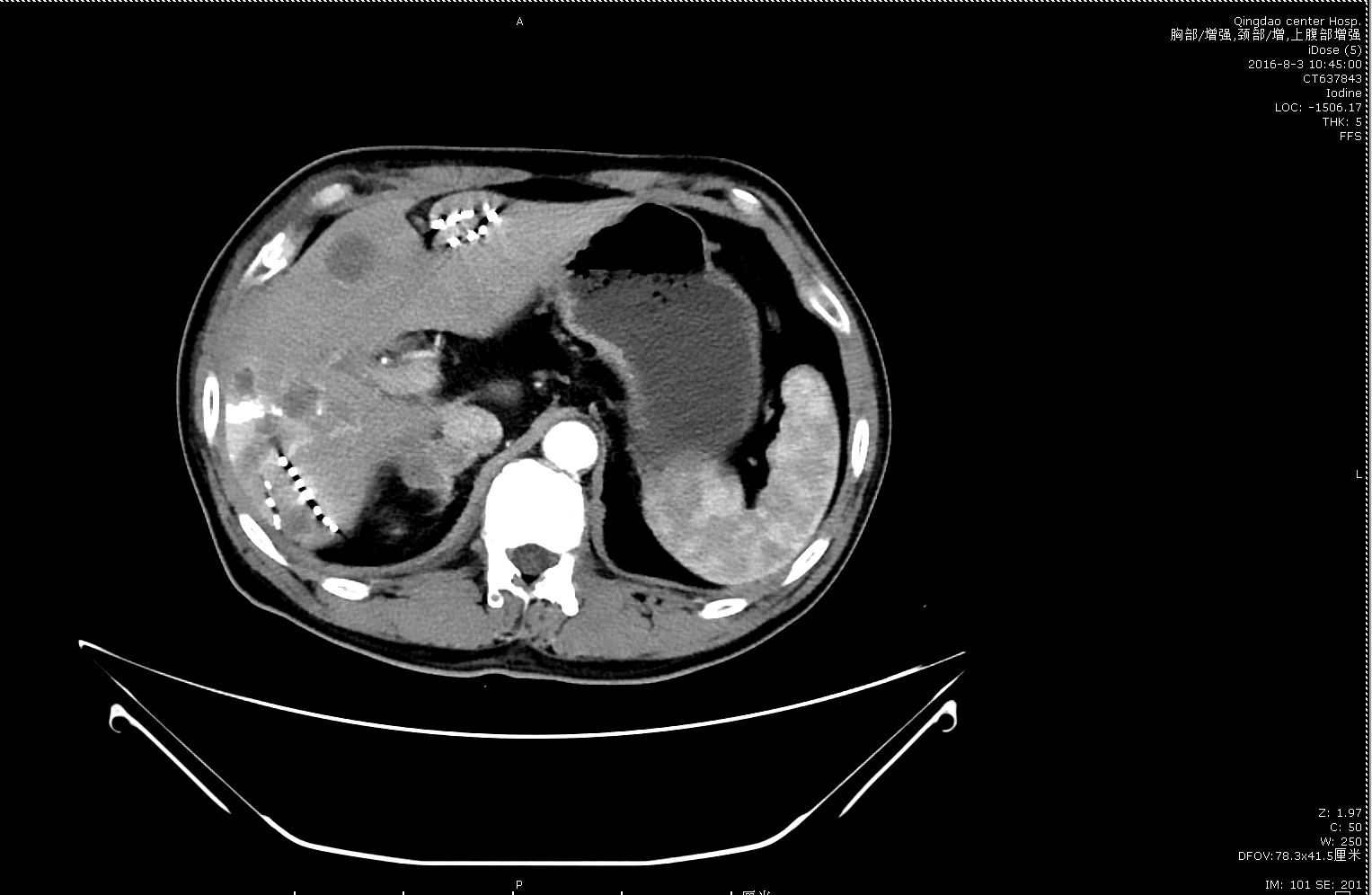

Supplement: Supplementary file 1 [file DataSheet_1.zip › original image/2016-8-3 Fig1.JPG]

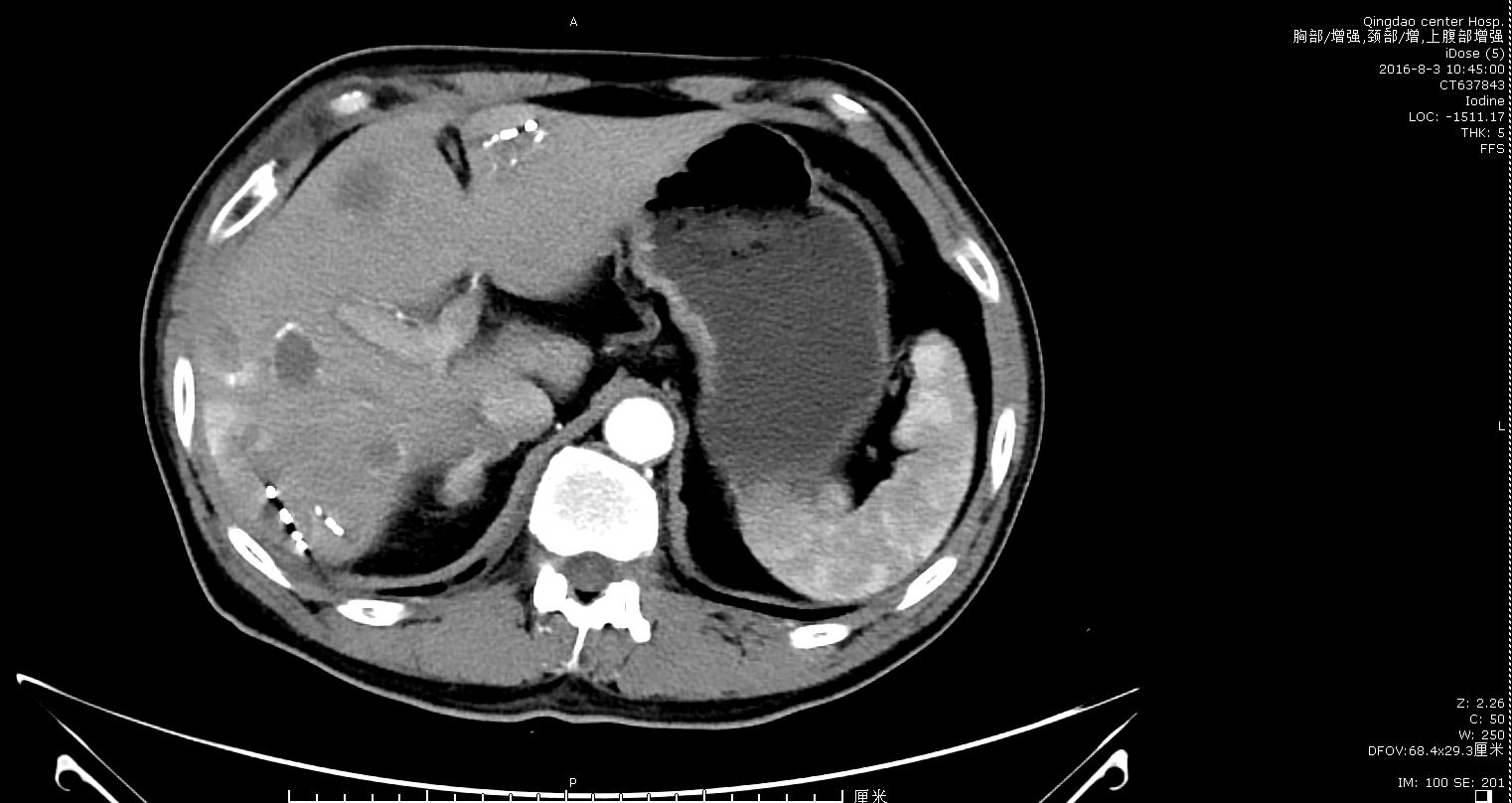

Supplement: Supplementary file 1 [file DataSheet_1.zip › original image/2016-8-3 Fig2.JPG]

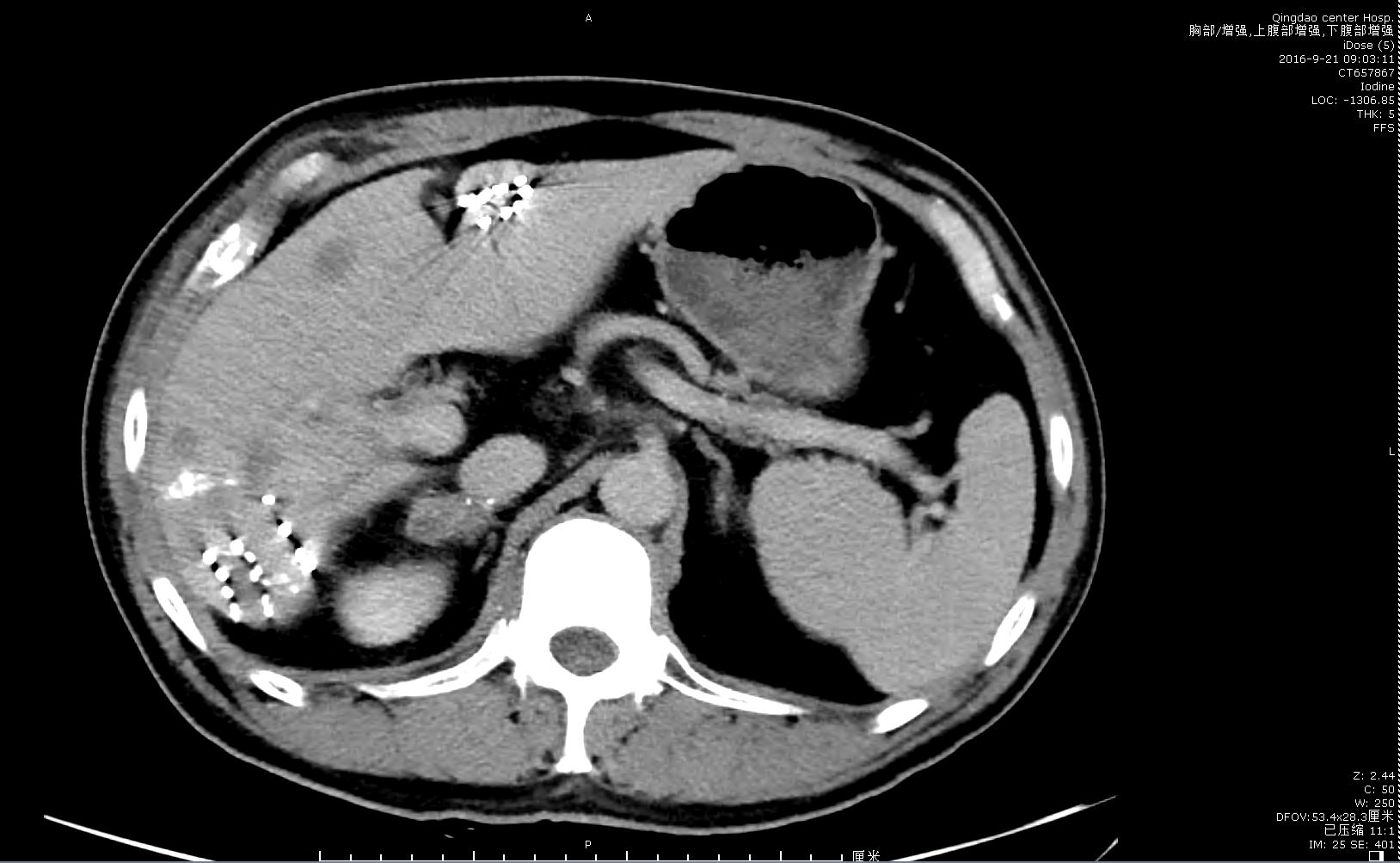

Supplement: Supplementary file 1 [file DataSheet_1.zip › original image/2016-9-21 Fig1.JPG]

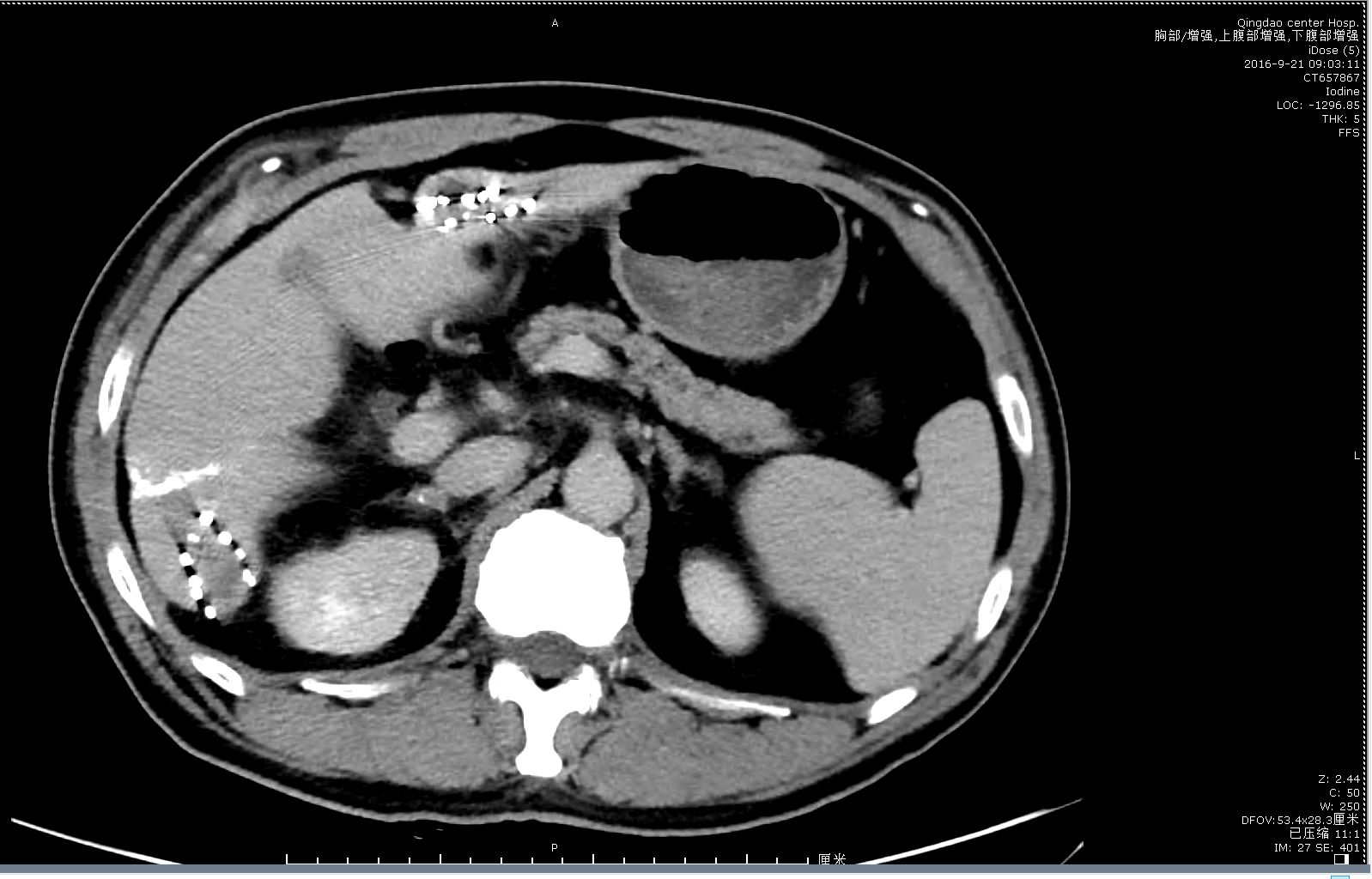

Supplement: Supplementary file 1 [file DataSheet_1.zip › original image/2016-9-21 Fig2.JPG]

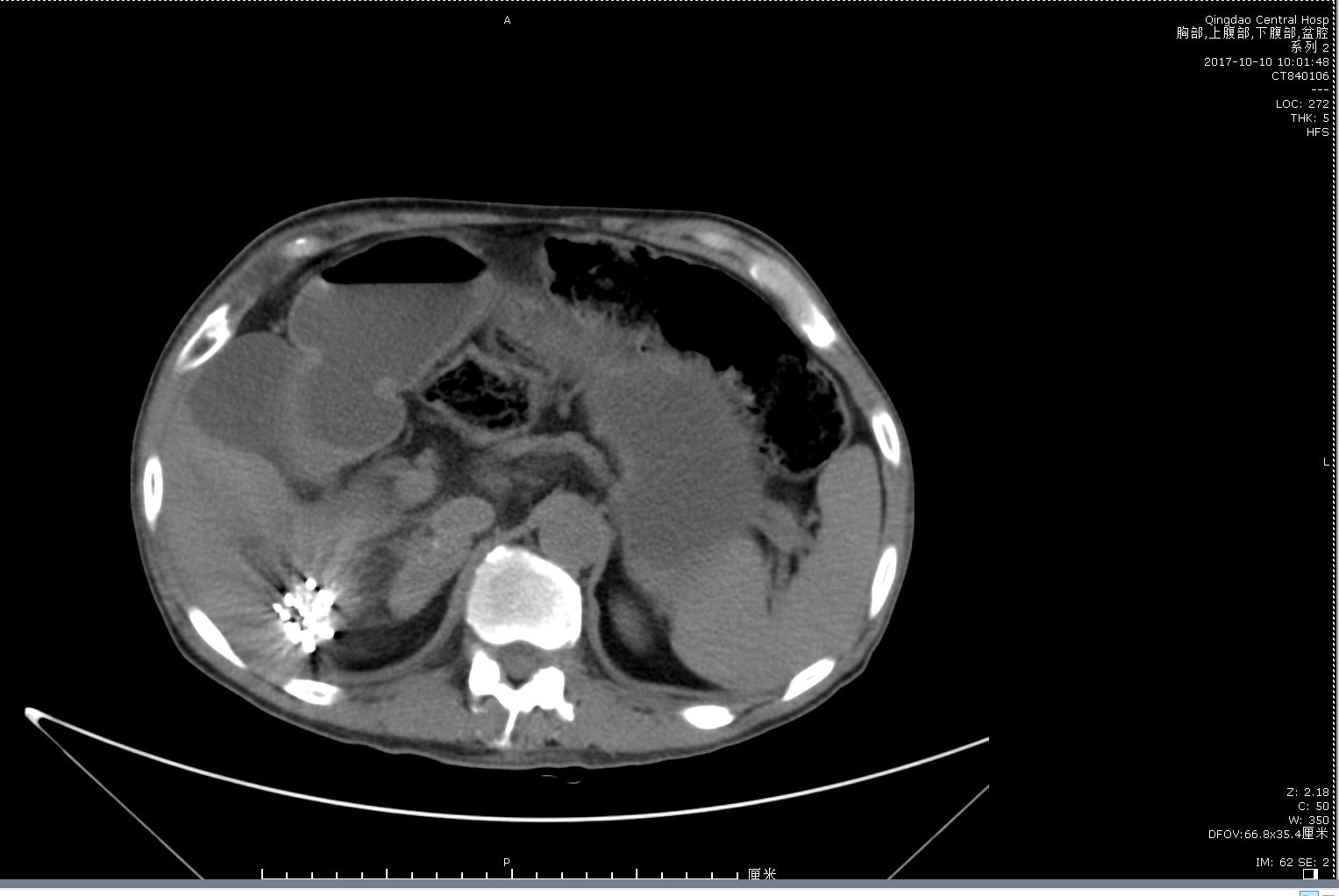

Supplement: Supplementary file 1 [file DataSheet_1.zip › original image/2017-10-10 Fig1.JPG]

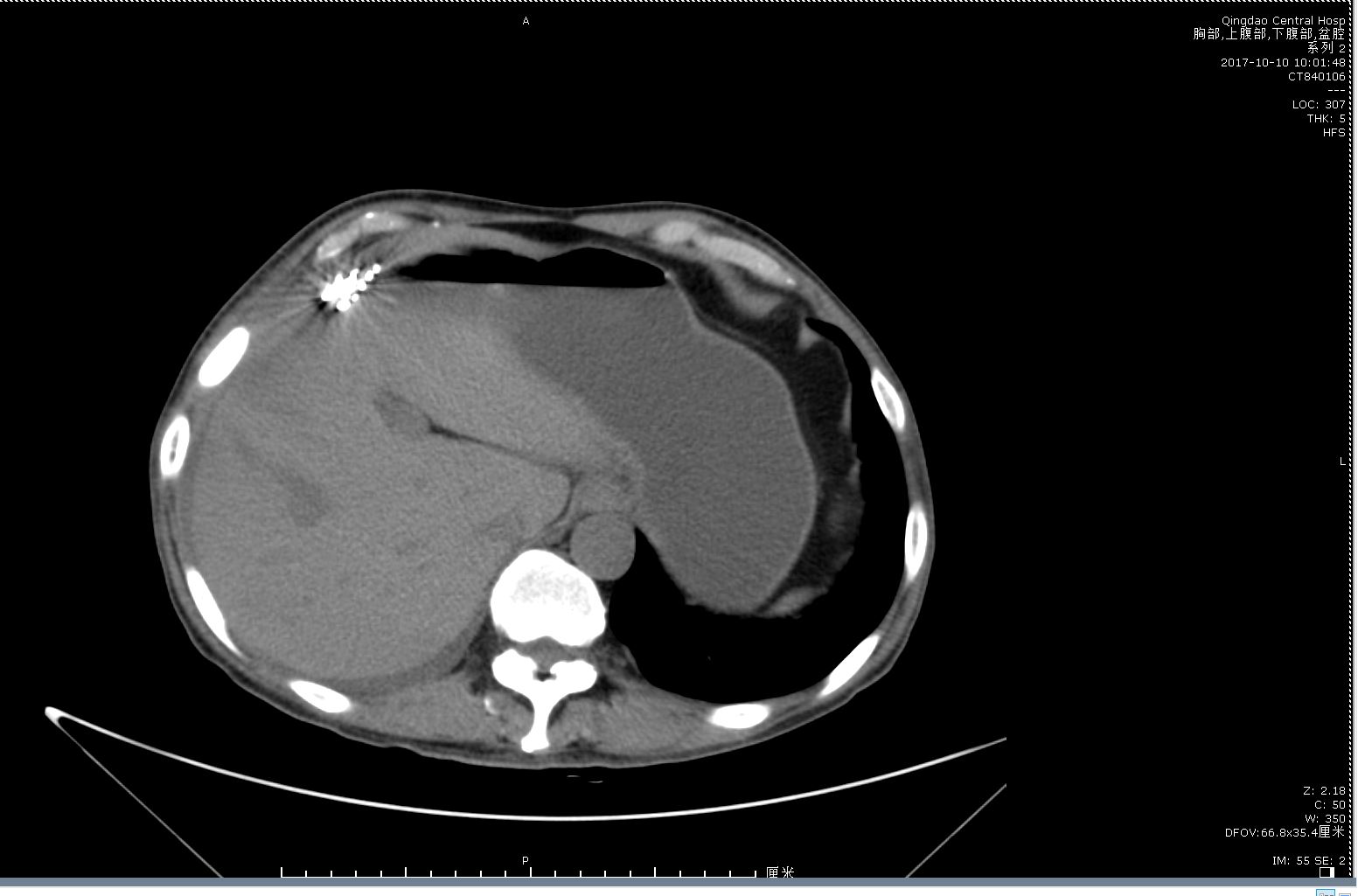

Supplement: Supplementary file 1 [file DataSheet_1.zip › original image/2017-10-10 Fig2.JPG]

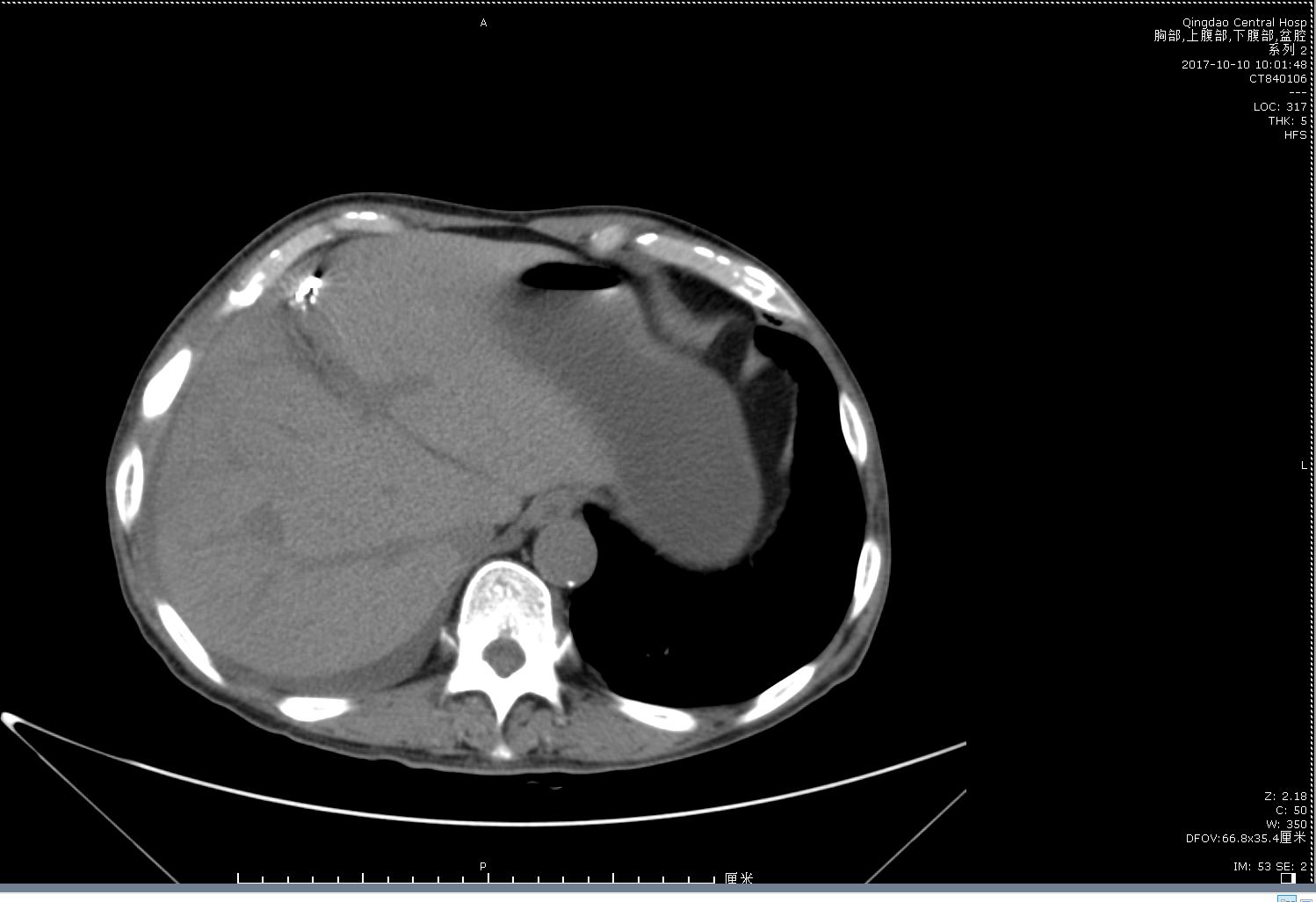

Supplement: Supplementary file 1 [file DataSheet_1.zip › original image/2017-10-10 Fig3.JPG]

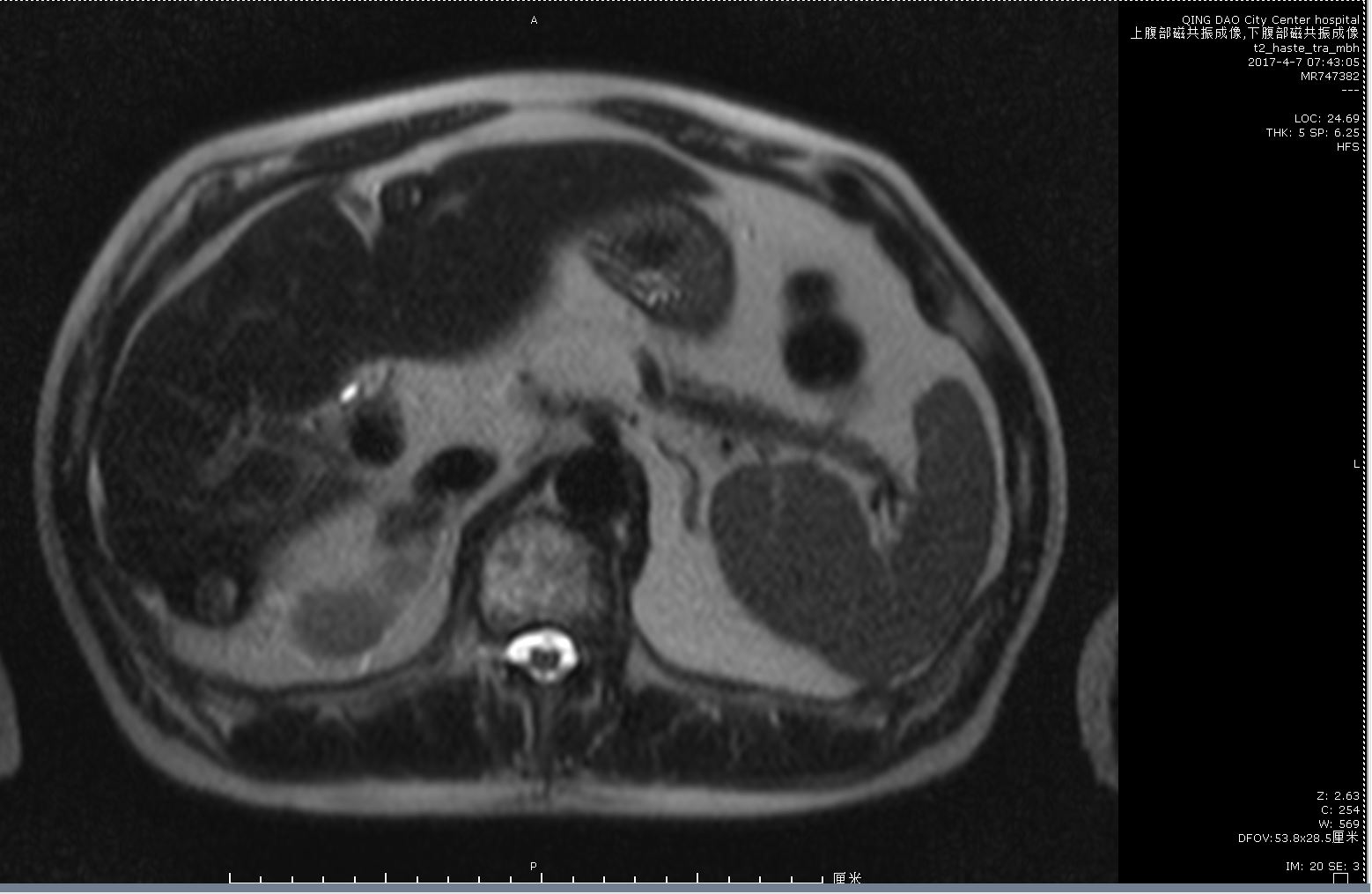

Supplement: Supplementary file 1 [file DataSheet_1.zip › original image/2017-4-7 Fig1.JPG]

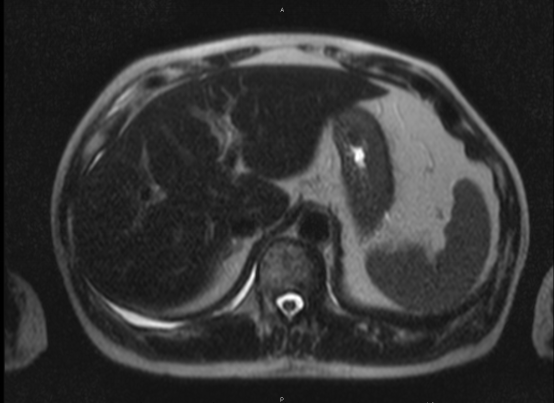

Supplement: Supplementary file 1 [file DataSheet_1.zip › original image/2017-4-7 Fig2.jpg]

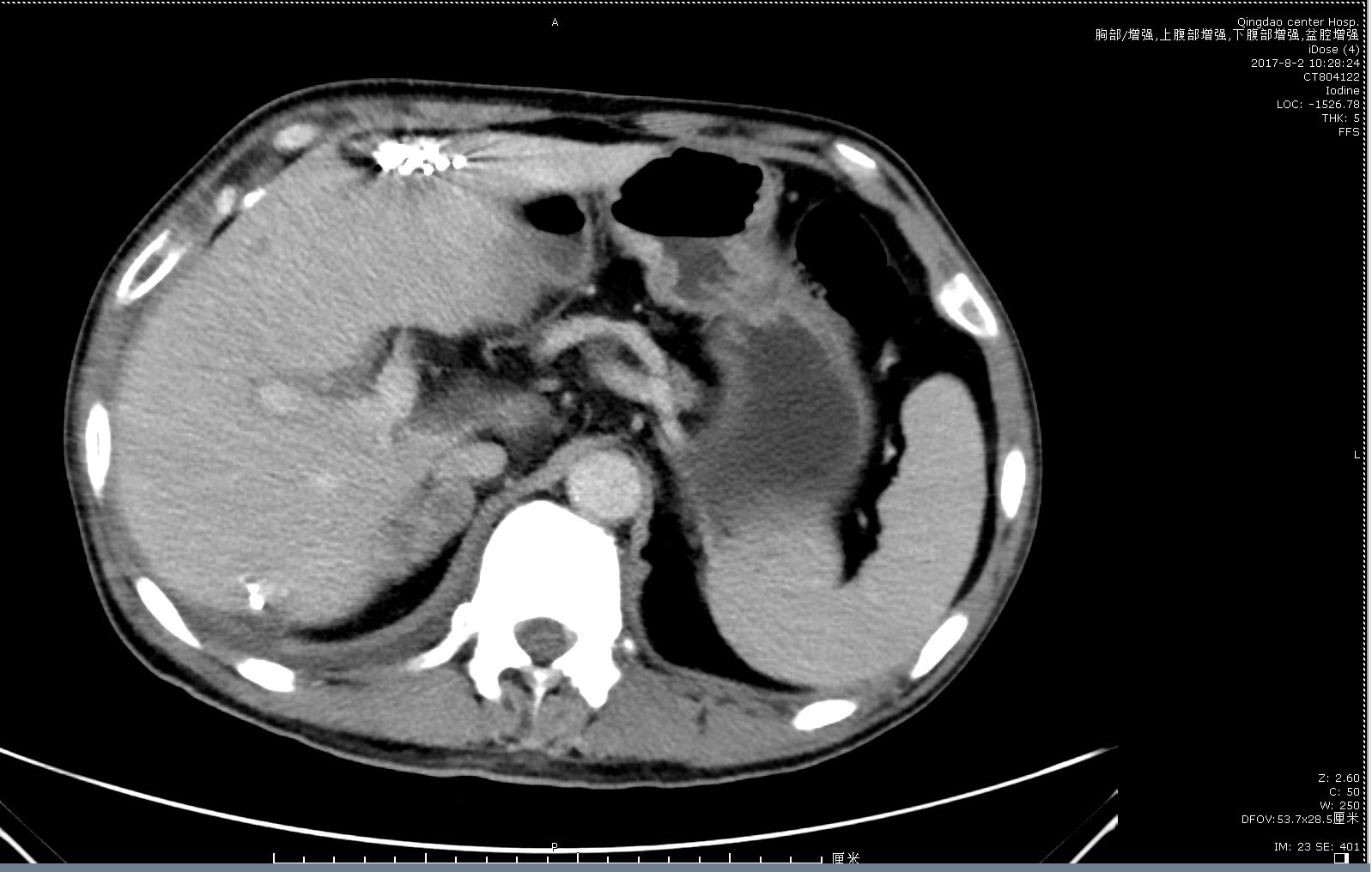

Supplement: Supplementary file 1 [file DataSheet_1.zip › original image/2017-8-2 Fig1.JPG]

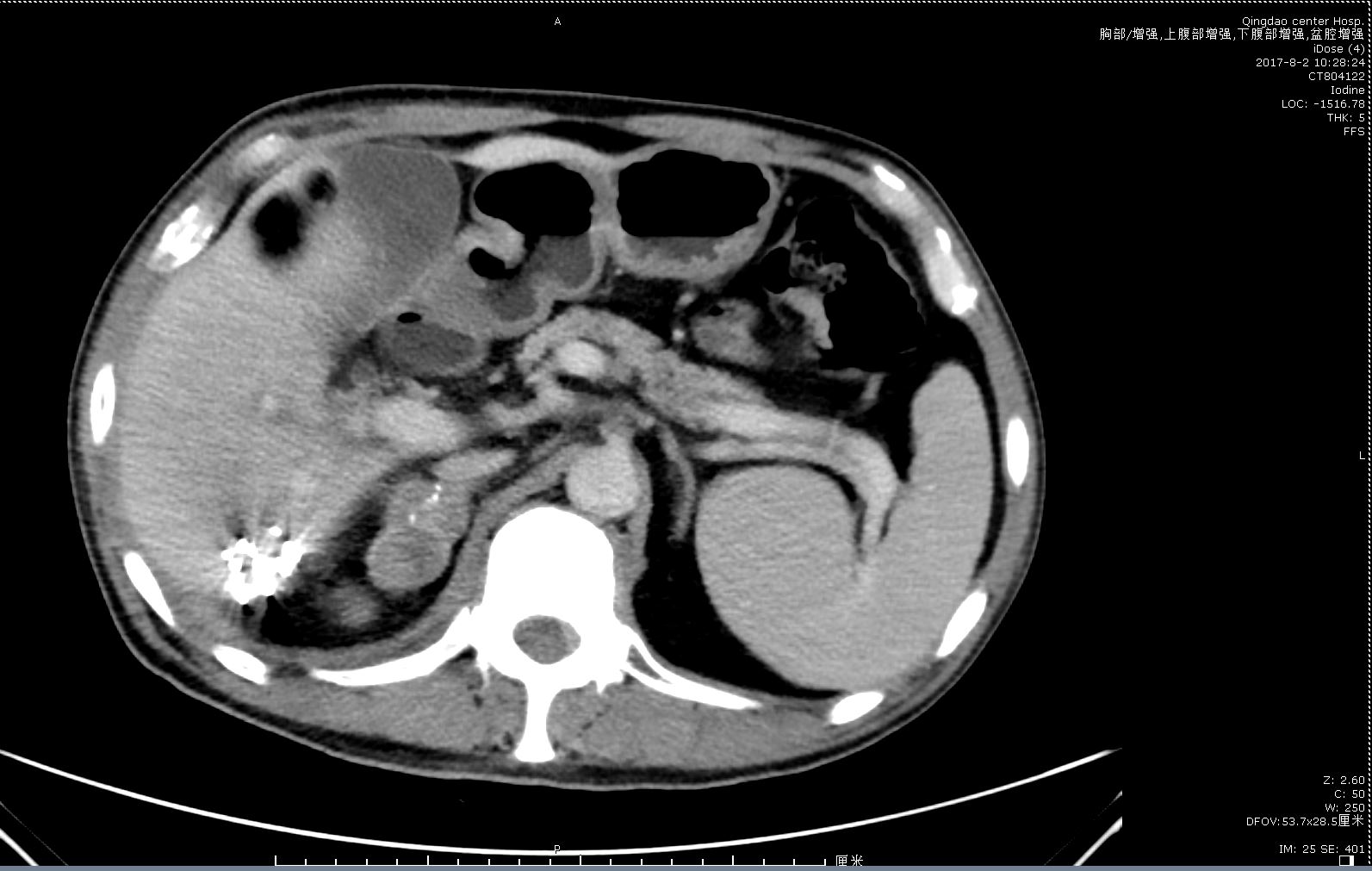

Supplement: Supplementary file 1 [file DataSheet_1.zip › original image/2017-8-2 Fig2.JPG]

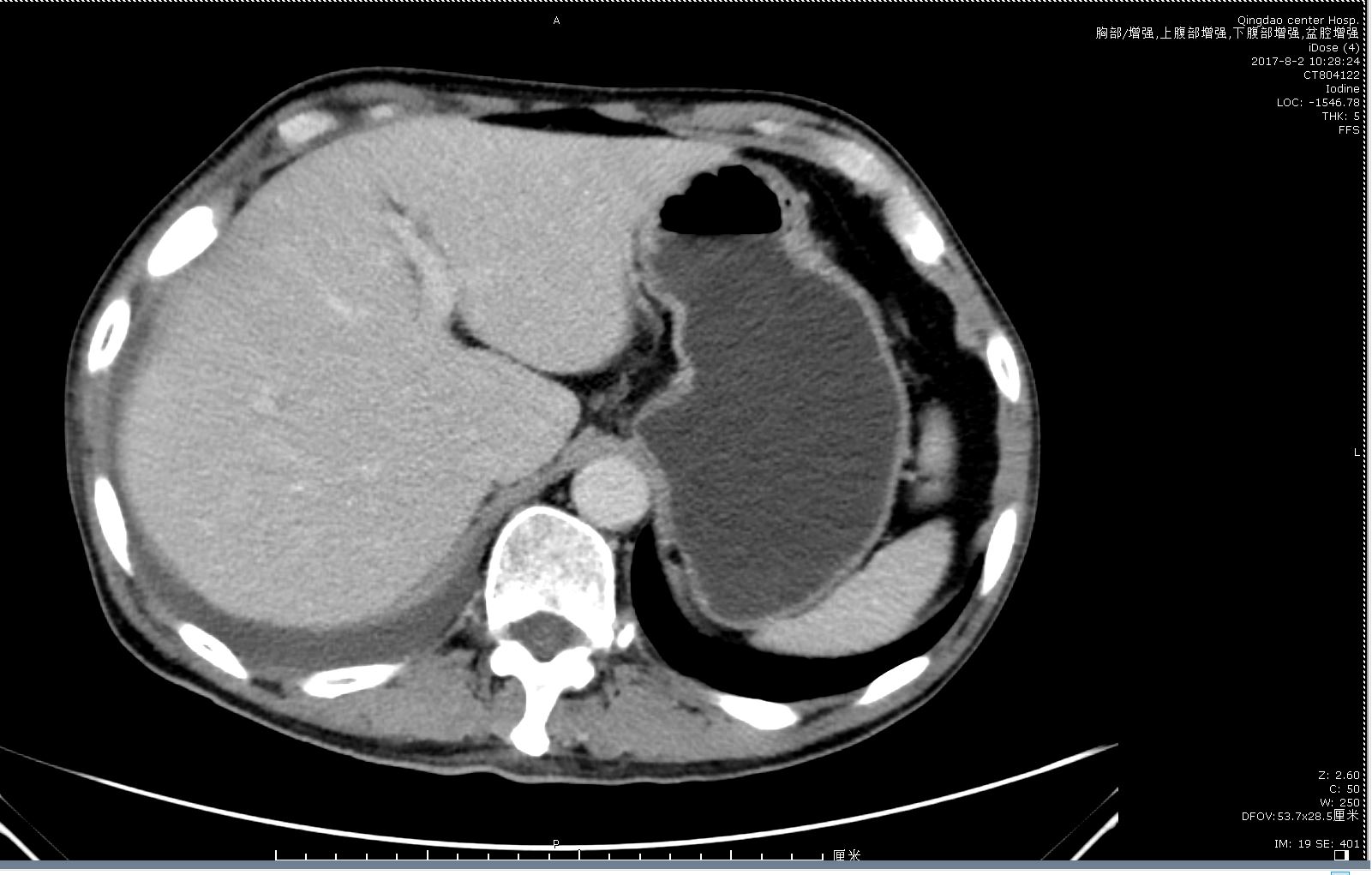

Supplement: Supplementary file 1 [file DataSheet_1.zip › original image/2017-8-2 Fig3.JPG]
